# Long Non-coding RNA *LINC00941* as a Potential Biomarker Promotes the Proliferation and Metastasis of Gastric Cancer

**DOI:** 10.3389/fgene.2019.00005

**Published:** 2019-01-22

**Authors:** Haiming Liu, Nan Wu, Zhe Zhang, XiaoDan Zhong, Hao Zhang, Hao Guo, Yongzhan Nie, Yuanning Liu

**Affiliations:** ^1^College of Computer Science and Technology, Jilin University, Changchun, China; ^2^State Key Laboratory of Cancer Biology, National Clinical Research Center for Digestive Diseases, Xijing Hospital of Digestive Diseases, Fourth Military Medical University, Xi’an, China; ^3^College of Life Sciences, Northwest University, Xi’an, China; ^4^Department of Pediatric Oncology, The First Hospital of Jilin University, Changchun, China

**Keywords:** gastric cancer, non-coding RNA, *LINC00941*, biomarker, TCGA

## Abstract

Gastric cancer (GC) is a considerable global health burden. Accumulating evidence suggests that long non-coding RNAs (lncRNAs) are aberrantly expressed in many cancers and play important roles in GC. However, only a few lncRNAs have been functionally characterized. In this study, we identified that long intergenic non-protein coding RNA 941 (*LINC00941*) is a potential biomarker for diagnosis and prognosis from the cancer genome atlas (TCGA), and we found that the expression of *LINC00941* is associated with tumor depth and distant metastasis in GC. Furthermore, functional enrichment analysis of *LINC00941* co-expression network demonstrated that *LINC00941* might be an essential regulator of tumor metastasis and cancer cell proliferation. To validate our findings, we utilized the loss-of-function analysis to reveal the biological function of *LINC00941* in GC cells. Loss-of-function analysis revealed that silence of *LINC00941* inhibits GC cells proliferation, migration, and invasion *in vitro* and modulates tumor growth *in vivo*. Our findings confirmed that *LINC00941* plays an important oncogenic function in GC and may serve as a potential biomarker for diagnosis and prognosis of GC.

## Introduction

Gastric cancer (GC) is one of the most frequently diagnosed cancer and the leading cause of cancer death in the world (Bray et al., 2018). In China, GC is a considerable health burden (Chen W. et al., 2018). The outlook for patients with metastatic GC is unfavorable, with median survival does usually not exceed 1 year (Van Cutsem et al., 2016). It is urgent to explore novel biomarkers and therapeutic targets for GC.

Long non-coding RNAs (lncRNAs) is an important subclass of non-coding RNAs (ncRNAs) and usually longer than 200 nucleotides with lack of protein-coding capability (Engreitz et al., 2016). Mounting evidence confirmed that lncRNAs are aberrantly expressed in many cancers and play key roles in promoting tumor initiation and progression (Lin and Yang, 2018). Several lncRNAs such as *UCA1*, *MALAT1*, *HOXA11-AS*, and *ZEB1-AS1* have been proposed as individual diagnostic or prognostic biomarkers in GC (Sun et al., 2016; Li et al., 2017a,b; Wang et al., 2017). However, only a few lncRNAs have been functionally characterized and the mechanism by which lncRNAs regulate GC remains to be fully elucidated.

Long intergenic non-protein coding RNA 941 (*LINC00941*), also known as MSC-upregulated factor (*lncRNA-MUF*), is an lncRNA located in the 12p11.21 region of the human genome. In hepatocellular carcinoma, high expressed *LINC00941* significantly promoted epithelial to mesenchymal transition (EMT) and malignant capacity (Yan et al., 2017). In lung adenocarcinoma, *LINC00941* displayed prognostic values and regulated PI3K-AKT signaling pathway (Wang et al., 2018). In GC, *LINC00941* is highly expressed in GC tissues and may participate in the process of GC (Luo et al., 2018). However, the precise biological function of *LINC00941* in GC has not been characterized.

In this study, we confirmed that *LINC00941* plays an important oncogenic function in GC by systematically integrating bioinformatics methods and *in vitro*/*vivo* studies. We identified that *LINC00941* acts as a potential diagnostic and prognostic biomarker in GC, and the expression of *LINC00941* is associated with tumor depth and distant metastasis based on the analysis of RNA-seq data from TCGA. In order to characterize the function of *LINC00941* in GC, we applied weighted gene co-expression network analysis (WGCNA) (Langfelder and Horvath, 2008) to constructed *LINC00941* co-expression network. Furthermore, through the functional enrichment analysis, we found that *LINC00941* might be an essential regulator of tumor metastasis and cancer cell proliferation. To validate our findings, we utilized the loss-of-function analysis to reveal the biological function of *LINC00941* in GC cells. Loss-of-function analysis confirmed that silence of *LINC00941* inhibits GC cells proliferation, migration, and invasion *in vitro* and modulates tumor growth *in vivo*. Our results demonstrated that *LINC00941* plays an important oncogenic function in GC and acts as a potential biomarker for diagnosis and prognosis of GC.

## Materials and Methods

### GC Data Collection and Processing

Gastric cancer data, including clinical information and gene expression data, were obtained from TCGA^1^ (Cancer Genome Atlas Research and Network, 2014). In this study, all samples with follow-up time exceeding 2000 days were excluded. GC data was processed as described in our previous work (Liu H. et al., 2018).

### Identifying Cancer-Related, Metastasis-Related, and Survival-Related lncRNAs

In order to identify cancer-related lncRNAs, we utilized Mann–Whitney U-test to compare the expression values between cancer samples and normal samples. To identify metastasis-related lncRNAs, we used the same statistical method to compare the expression values between metastatic (M1) samples and non-metastatic (M0) samples. The cancer-related and metastasis-related lncRNAs with *p*-values < 0.05 and fold change ≥ 1 were considered significant. In order to identify survival-related lncRNAs, we divided samples into two groups (high and low) based on the median expression level of each lncRNA. We then utilized univariate Cox proportional-hazards regression model and log-rank test to identify survival-related lncRNAs. LncRNAs with *p* < 0.05 were considered survival related. Kaplan-Meier plot was used to represent the results.

### LncRNA Co-expression Network Construction and Functional Enrichment Analysis

Long non-coding RNAs co-expression network construction was performed by combining lncRNAs with genes expression data using the R package WGCNA (Langfelder and Horvath, 2008). First, the expression matrix was constructed based on the Spearman’s rank correlation coefficient between all gene pairs. Then, the expression matrix was converted into an adjacency matrix (AM), and AM was further converted into a topological overlap matrix (TOM). Based on TOM, the average-linkage hierarchical clustering method was used to cluster the genes and dynamic tree cut algorithm was used to identify lncRNA co-expressed module that was set the minimum module size to 30. To determine the functions of lncRNA co-expressed module, the genes in the module were subjected to functional enrichment analysis by gene ontology (GO) (Antonazzo et al., 2017) and Kyoto Encyclopedia of Genes and Genomes (KEGG) (Kanehisa et al., 2017) analyses with DAVID 6.8^2^ (Huang da et al., 2009). In this study, the result of functional enrichment analysis with FDR < 0.05 was considered statistically significant.

### Cell Culture, RNA Extraction, and Real-Time PCR

The human GC cells (MKN45 and AGS) were purchased from American Type Culture Collection (ATCC). Cell culture, RNA extraction, and real-time PCR were performed as described in our previous work (Liu H. et al., 2018). Primer sets specific for *LINC00941* were designed by RiboBio (RiboBio, China). The primer sequences of *LINC00941* were as follows: *LINC00941* forward, 5′- ACCACTACACTCAGCCAAATAC-3′, reverse, 5′ - GGCTATCAACTGTCTCCTTTAGAC-3′.

### Cell Transfection

The short interfering RNA (siRNA) targeting *LINC00941* were synthesized by RiboBio (RiboBio, China). The siRNA sequences for *LINC00941* were as follows: LINC0 0941-si1: 5′- ACGCGTTGCATAACCTGA-3′, LINC00941-si2: 5′- GAGACAGTTGATAGCCAAA-3′. Oligonucleotide transfection was performed using Lipofectamine 2000 reagent (Invitrogen, United States). Short hairpin RNA (shRNA) directed against *LINC00941* were synthesized by GeneChem (Shanghai, China) and were inserted into the vector. The sequence of the effective shRNA was as follows: sh-LINC00941: GAGACAGTTGATAGCCAAA. We utilized an empty vector as a negative control (VECTOR).

### CCK-8, Colony Formation, Cell Migration, and Invasion Assays

CCK-8 and colony formation assays were performed as described in our previous work (Liu H. et al., 2018). Cell migration and invasion assays were measured using Transwell chamber (Corning, United States) with 8-μm pore membrane. Cells need to be starved for 12 h in serum-free medium before the experiment. For the migration assays, 5 × 10^5^ cells were seeded into the top chamber of transwell. For the invasion assays, 1 × 10^5^ cells were seeded top chamber of transwell with Matrigel (Corning, United States). After 24 h, non-invasive cells were removed, and that migrated or invaded cells were fixed and stained with 5% crystal violet (Beyotime Biotechnology, China). Cells were photographed and counted in five random fields at 100× magnification.

### Protein Extraction and Western Blot Analysis

Cells were washed three times with PBS and collected in RIPA lysis buffer (Beyotime Biotechnology, China) supplemented with a protease inhibitor cocktail (Calbiochem, United States). Protein concentration was determined by staining with Coomassie Blue (Beyotime Biotechnology, China). After electrophoresis, the protein was transferred to a polyvinylidene fluoride membrane (Merck Millipore, Germany). After blocking with 0.1% Tween 20 (TBS-T) in Tris-buffered saline containing 5% skim milk for 1 h at room temperature, the primary monoclonal antibody was added to the membrane and incubated overnight at 4°C. The next day, the membrane was incubated with the corresponding secondary antibody for 1 h at room temperature and the signal was detected in a Bio-Rad ChemiDoc XRS imaging system. The ratio of the gray value of the target protein to the gray value of β-actin indicates the relative amount of protein. Primary antibodies were used as follows: anti-E-cadherin (1:1000; Cell Signaling Technology, United States), anti-Fibronectin (Cell Signaling Technology, United States) and anti-Snail1 (Cell Signaling Technology, United States), and anti-GAPDH (Santa Cruz Biotech, United States).

### Tumorigenicity Assays in Nude Mice

Empty vector (negative control) and sh-LINC00941 stained cells SGC-7901 were injected subcutaneously into either side of the axillary region of male BALB/c nude mice (4–5 weeks old). Ten female BALB/c nude mice (5–6 weeks old) were randomly divided into two groups (sh-LINC00941 and VECTOR) and the mice were maintained under specific pathogen-free conditions. To establish the subcutaneous xenograft tumor model, 1 × 10^7^ cells were injected into the right side of the back of nude mice. We measured and recorded nude mice body weight and tumor size weekly. After 4 weeks, all the nude mice were sacrificed under deep anesthesia. This study was carried out in strict accordance with the ethical standards of the Fourth Military Medical University. The tumor volumes were calculated using the following formula: tumor volume (mm^3^) = [length (mm) × width^2^ (mm) × π ]/6.

## Results

### *LINC00941* Is a Potential Biomarker for Diagnosis and Prognosis of GC

**FIGURE 1:**
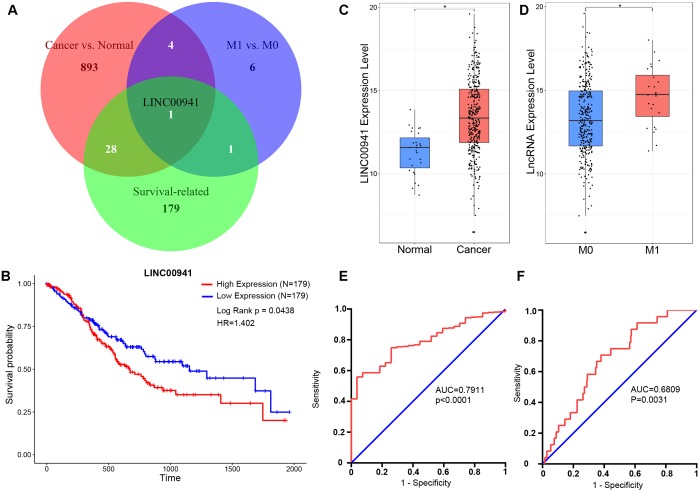
*LINC00941* is potential diagnosis and prognosis biomarker for GC. **(A)** Venn diagram showed that only *LINC00941* presented in the overlap of three data sets. **(B)** High expression of *LINC00941* was associated with poor prognosis in GC. **(C)** *LINC00941* were up-regulated in cancer samples, ^∗^*p* < 0.05. **(D)** *LINC00941* was significantly up-regulated in M1 samples, ^∗^*p* < 0.05. **(E)** The ROC curve analysis for discriminative ability between cancer samples and normal samples. **(F)** The ROC curve analysis for discriminative ability between M1 samples and M0 samples.

We obtained 385 samples with gene expression profiles and clinical information from TCGA. By comparing 358 cancer samples with 27 normal samples, we identified 926 significantly cancer-related lncRNAs (fold change ≥ 1, *p* < 0.05, Mann-Whitney U-test, Supplementary Table S1). By comparing 24 metastatic (M1) samples with 316 non-metastatic (M0) samples, we identified 12 significantly metastasis-regulated lncRNAs (fold change = 1, *p* < 0.05, Mann-Whitney U-test, Supplementary Table S2). Then, we found 209 survival-related lncRNAs using univariate Cox proportional-hazards regression model (HR > 1, *p* < 0.05, log-rank test, Supplementary Table S3). As shown in Figure 1A, we identified that only *LINC00941* is a survival-related (Figure 1B), cancer-related (Figure 1C), and metastasis-related lncRNA (Figure 1D). In addition, receiver operating characteristic (ROC) curve analysis was utilized to explore whether the expression level of *LINC00941* could discriminate samples, and area under the ROC curve (AUC) was performed to calculate the diagnostic sensitivity and specificity. As shown in Figures 1E,F, the expression of *LINC00941* discriminated cancer samples from normal samples (AUC = 0.7911, 95% CI: 0.7264–0.8559, *p* < 0.0001) and discriminated M1 samples from M0 samples (AUC = 0.6809, 95% CI: 0.5852–0.7766, *p* = 0.0031). Our findings indicated that *LINC00941* is a potential biomarker for diagnosis and prognosis of GC.

### The Expression of *LINC00941* Is Associated With Tumor Depth and Distant Metastasis of Patients in GC

To explore the clinical value of LINC00941, Mann-Whitney U-test and Chi-square test were used to compare the different clinicopathological features in each group according to the expression of *LINC00941* (Table 1 and Supplementary Table S4). The aberrantly expressed *LINC00941* was observed in several clinicopathological features, such as tumor depth (*p* = 0.018, Mann-Whitney U-test), lymph node metastasis (*p* = 0.0184, Mann-Whitney U-test), distant metastasis (*p* = 0.003, Mann-Whitney U-test), and Tumor stage (*p* = 0.042, Mann-Whitney U-test). The results of chi-square test analysis demonstrated that the expression of *LINC00941* had no significant correlation with clinicopathological features including gender, histological type, neoplasm histologic grade, lymph node metastasis, and tumor stage. However, the expression of *LINC00941* expression was associated with tumor depth (*p* = 0.0197, chi-squared test) and distant metastasis (*p* = 0.0111, chi-squared test). By combining the results of two test methods, our results indicated that the expression of *LINC00941* was associated with tumor depth and distant metastasis of patients in GC.

**Table 1:** Associations between the expression of *LINC00941* and clinicopathological features in GC.

Clinicopathological features	No. of patients		*LINC00941* expression			
		M ± *SD*	*P*-value (u test)	HIGH	LOW	*P*-value (chi-squared test)

**Gender**						
Male	229	13.2948 ± 2.1672	0.075	108	121	0.1524
Female	129	13.7071 ± 2.2679		71	58	
Histological type						
Intestinal	157	13.4669 ± 2.2533	0.324	83	74	0.1936
Diffuse	69	13.1354 ± 1.9947		30	39	
**Neoplasm histologic grade**						
G1+G2	138	13.3593 ± 2.2462	0.722	67	71	0.6931
G3	211	13.5146 ± 2.1727		107	104	
**Pathological T**						
I/II	89	13.0108 ± 2.1219	0.018^∗^	35	54	0.0197^∗^
III/IV	261	13.5589 ± 2.2355		140	121	
**Pathological N**						
N0	104	13.089 ± 2.2773	0.0184^∗^	45	59	0.0994
N1-3	236	13.6186 ± 2.1909		125	111	
**Pathological M**						
M0	316	13.3147 ± 2.2169	0.003^∗^	152	164	0.0111^∗^
M1	24	14.6567 ± 1.7331		18	6	
**Tumor stage**						
Stage I–II	154	13.202 ± 2.2414	0.042^∗^	70	84	0.1129
Stage III–IV	181	13.6303 ± 2.2126		98	83	


*^∗^The values had statistically significant differences.*

*M, the average expression level; SD, the standard deviation of expression level; Pathological T, tumor depth; Pathological N, lymph nodes affected; Pathological M, distant metastasis.*

### Construction and Functional Enrichment Analysis of *LINC00941* Co-expression Network

**FIGURE 2:**
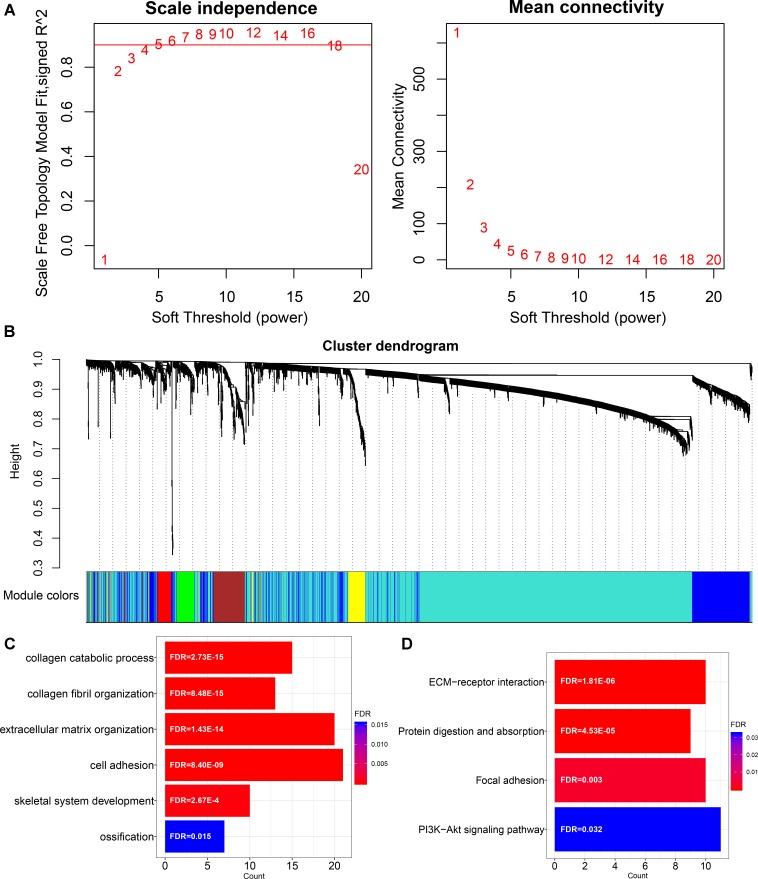
Construction and functional enrichment analysis of *LINC00941* co-expression network. **(A)** Scale-free topology index and mean connectivity were used to determine the soft threshold. **(B)** Clustering dendrogram of *LINC00941* co-expression network. The genes are assigned to different modules that are distinguished by different colors. **(C)** The GO terms in *LINC00941* co-expression model in GC. **(D)** The KEGG pathways in *LINC00941* co-expression model in GC.

In order to characterize the function of *LINC00941* in GC, we applied WGCNA to constructe *LINC00941* co-expression network by combining *LINC00941* with dysregulated coding genes in GC samples (absolute fold change ≥ 1, *p* < 0.05, Mann-Whitney U-test, Supplementary Table S5). We selected the soft threshold power (β = 4) to ensure that the co-expression network was scale-free topology (Figure 2A). After hierarchical clustering and dynamic tree cutting, a total of 28 co-expression modules were identified (Figure 2B). We found that *LINC00941* is clustered in “yellow” module, which contained 123 dysregulated coding genes (Supplementary Table S6). The genes in *LINC00941* co-expression module was applied to explore the function of *LINC00941* via the functional enrichment analysis. In this study, we identified 6 GO terms in BP and 4 KEGG pathways (Figures 2C,D and Supplementary Table S7). Some GO terms, such as extracellular matrix organization (GO: 0030198) and cell adhesion (GO: 0007155) are cell migration-related GO processes, which are associated with tumor metastasis. We detected that ECM-receptor interaction (KEGG: hsa04512) and Focal adhesion (KEGG: hsa04510) are metastasis-related pathways. PI3K-Akt signaling pathway (KEGG: hsa04151) is cell proliferation-related pathway. Our findings demonstrated that *LINC00941* could be a potential regulator of tumor metastasis and cancer cell proliferation.

### Silence of *LINC00941* Inhibits GC Cells Proliferation *in vitro*

**FIGURE 3:**
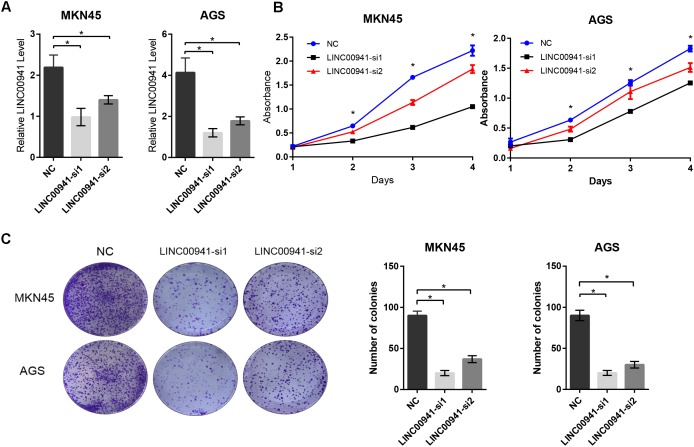
Silence of *LINC00941* inhibits GC cells proliferation *in vitro*. **(A)** *LINC00941* was knocked down by siRNAs in MKN45 and AGS cells. **(B)** Silence of *LINC00941* significantly inhibited the proliferation ability of MKN45 and AGS cells. The effect on cell proliferation was assessed over 4 days using the CCK8. **(C)** Colony formation ability of MKN45 and AGS cells was significantly decreased after silencing *LINC00941*. Two-tailed Student’s *t*-test was used to analyze the significant differences, ^∗^*p* < 0.05.

To validate our findings, we designed two siRNAs, which can specifically target *LINC00941*. As shown in Figure 3A, *LINC00941* was effectively knocked down by siRNAs in MKN45 and AGS cells. Then, we assessed whether silence of *LINC00941* could affect the proliferation ability of MKN45 and AGS cells. CCK8 assay indicated that silence of *LINC00941* significantly inhibited proliferation ability of MKN45 and AGS cells (Figure 3B). As shown in Figure 3C, colony formation ability of MKN45 and AGS cells was significantly decreased after silencing *LINC00941*. Our results confirmed that *LINC00941* promotes GC cells proliferation *in vitro*.

### Silence of *LINC00941* Reduces GC Cells Migration and Invasion *in vitro*

**FIGURE 4:**
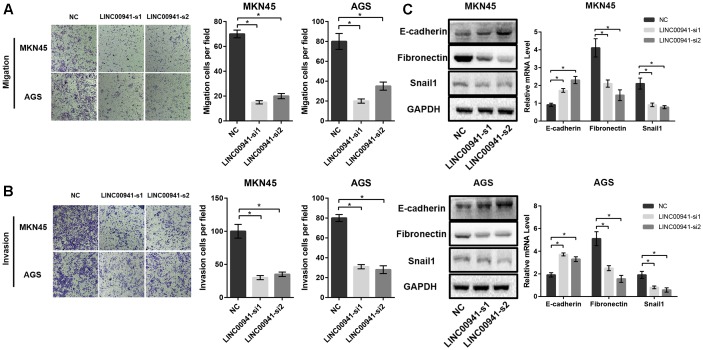
Silence of *LINC00941* inhibits GC cells migration and invasion *in vitro*. **(A)** Silence of *LINC00941* inhibits the migration abilities of MKN45 and AGS cells. **(B)** Silence of *LINC00941* inhibits the invasive ability of MKN45 and AGS. **(C)** The expression of E-cadherin, Fibronectin, and Snail1 in MKN45 and AGS cells was assessed by western blot and qRT-PCR. *GAPDH* was used as a loading control. The expression was normalized to *GAPDH*. Two-tailed Student’s *t*-test was used to analyze the significant differences, ^∗^*p* < 0.05.

To further analyze the effect of *LINC00941* on GC metastasis, we utilized cell migration and invasion assays to explore the effect of silencing *LINC00941* on metastasis ability of MKN45 and AGS cells. The results of transwell migration assay indicated that silence of *LINC00941* inhibits the migration abilities of MKN45 and AGS cells (Figure 4A). The results of matrigel invasion assay indicated that silence of *LINC00941* also inhibited the invasion abilities of GC cells (Figure 4B). Epithelial cell mesenchymal transition (EMT) is a process that is associated with tumor metastasis and lncRNAs could modulate cancer metastasis via affecting EMT (Xu et al., 2016; Grelet et al., 2017; Min et al., 2017). To explore the association between *LINC00941* and EMT in GC metastasis, we measured the expression level of EMT markers, such as E-cadherin (*CDH1*), fibronectin (*FN*), and Snail1 (*SNAI1*), using western blot and qRT-PCR. The results confirmed that silence of *LINC00941* decreased the protein and gene expression of *CDH1*, while increased the expression of *FN* and *SNAI1* in MKN45 and AGS cells (Figure 4C). Therefore, our results demonstrated that *LINC00941* might modulate GC cells metastatic properties via affecting EMT biomarker and could regulate GC cells migration and invasion *in vitro*.

### Silence of *LINC00941* Suppresses Tumor Growth *in vivo*

**FIGURE 5:**
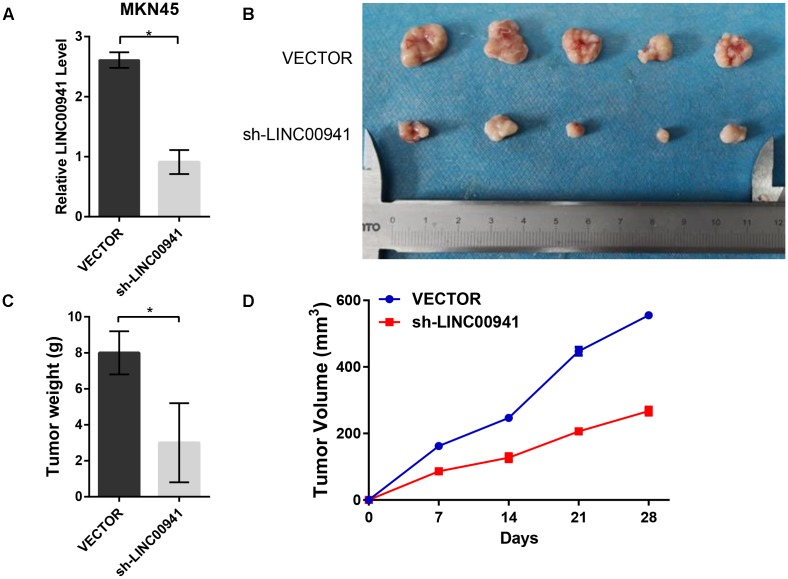
Silence of *LINC00941* suppresses tumor growth *in vivo*. **(A)** *LINC00941* was knocked down by shRNA in MKN45 cells. **(B)** The tumors in sh-LINC00941-infected nude mice significantly smaller than the negative control. **(C)** Tumor weights in nude mice with sh-LINC00941-infected were significantly lighter than the negative control. **(D)** Tumor volumes in nude mice with sh-LINC00941-infected significantly smaller than the negative control. Two-tailed Student’s *t*-test was used to analyze the significant differences, ^∗^*p* < 0.05.

To further validate the effect of *LINC00941* on tumor growth, we used stably expressing MKN45 cells by infection with lentivirus expressing sh-LINC00941 and negative control. As shown in Figure 5A, *LINC00941* was effectively knocked down by shRNA in MKN45 cells. We then evaluated the tumorigenic effects in BALB/c nude mice. The tumors in nude mice with sh-LINC00941-infected significantly smaller than the negative control (Figure 5B). We also measured the tumors weight and tumors volume of the two groups. We found that the tumor weight (Figure 5C) and the tumor volume (Figure 5D) of sh-LINC00941 were significantly smaller than the negative control. Thus, our findings confirmed that *LINC00941* could modulate tumor growth *in vivo*.

## Discussion

Non-coding RNAs represent more than 98% of the total human transcriptome (Yvonne et al., 2014) and the aberrantly expressed lncRNAs drive important cancer phenotypes (Schmitt and Chang, 2016). Few lncRNAs, such as *UCA1*, *MALAT1*, *HOXA11-AS*, and *ZEB1-AS1* have been reported to play important roles in GC. However, only several lncRNAs have been functionally characterized in GC.

In this study, we systematically integrated GC clinical information and gene expression data from TCGA. By comparing cancer samples with normal samples, we identified 926 cancer-related lncRNAs, such as *FEZF1-AS1* (Liu Y.W. et al., 2017), *HOTAIR* (Okugawa et al., 2014), *HOXA11-AS* (Sun et al., 2016), *HOTTIP* (Zhao et al., 2018), and *LINC01234* (Chen X. et al., 2018) have been reported to play important roles in GC. By comparing metastatic samples with non-metastatic samples, we identified 12 metastasis-related lncRNAs, such as *LINC00704* (Tracy et al., 2018), *LINC00460* (Li et al., 2018), and *LINC00520* (Henry et al., 2016) have been reported to associate with tumor metastasis. By using univariate Cox proportional-hazards regression model and ROC curve analysis, we identified that the *LINC00941* may serve as a potential biomarker for diagnosis and prognosis of GC. Furthermore, we explored the associations between expression of *LINC00941* and clinicopathological features, and we found that the expression of *LINC00941* was associated with tumor depth and distant metastasis.

To further explore the function of *LINC00941* in GC, *LINC00941* co-expression network was constructed by combining *LINC00941* with dysregulated coding genes in GC using R package WCGNA. After hierarchical clustering and dynamic tree cutting, *LINC00941* co-expression module was identified. The dysregulated coding genes in the module was applied to explore the function of *LINC00941* via the functional enrichment analysis. The results of GO terms and KEGG pathways were mainly enriched in cell proliferation, cell migration, and tumor metastasis. Our findings indicated that *LINC00941* might play an important oncogenic function in GC. *LINC00941* located in the 12p11.21 region of the human genome and also known as lncRNA-MUF. In lung adenocarcinoma, survival of patients was negatively associated with high expression of *LINC00941* which modulates focal adhesion and PI3K-AKT signaling pathway (Wang et al., 2018). In hepatocellular carcinoma, high expression of *LINC00941* significantly promoted EMT and malignant capacity tissues and correlated with poor prognosis (Yan et al., 2017). *LINC00941* was differentially expressed in TGF-β1-activated A549 cells compared with those in normal controls (Liu H. et al., 2017) and was up-regulated upon treatment of colon cancer cells with chemotherapeutic drugs (Zinovieva et al., 2018). In GC, *LINC00941* is up-regulated in tumor tissues (Luo et al., 2018). However, in GC, the precise biological function of *LINC00941* have not been characterized.

To validate our findings, we utilized the loss-of-function analysis to reveal the biological function of *LINC00941* in GC cells. CCK-8 and colony formation assays confirmed that *LINC00941* could promote GC cells proliferation. Cell migration and invasion assays confirmed that *LINC00941* could promote GC cells metastasis. Previous studies revealed that EMT is associated with tumor metastasis and lncRNAs could modulate cancer metastasis via affecting EMT (Xu et al., 2016; Grelet et al., 2017; Min et al., 2017). We further measured the expression level of EMT markers such as E-cadherin, Snail (Dong et al., 2012), and Fibronectin (Park and Schwarzbauer, 2014). The results demonstrated that *LINC00941* could regulate GC cells metastasis via affecting EMT. Therefore, *LINC00941* might modulate GC cells metastatic properties via affecting EMT biomarker. In this study, there are still some limitations and the specific biological mechanism by *LINC00941* acts still needs to be investigated.

In summary, by systematically integrating bioinformatics and experimental methods, our findings firstly revealed that *LINC00941* plays an important oncogenic function in GC. Our findings not only provide novel insights on the functional characterization of *LINC00941* in GC, but can also provide a novel biomarker for diagnosis and prognosis of GC in future studies.

## Ethics Statement

This study was carried out in accordance with the recommendations of the National Institutes of Health Laboratory Animal Care and Use Guidelines. The protocol was approved by the Xijing Hospital Institutional Review Board.

## Author Contributions

YL and YN conceived of and directed the project. HL, NW, and ZZ designed and performed the experiments. HL and HG conducted the data analysis and interpreted the results. XZ and HZ revised the manuscript critically for important intellectual content. HL and NW wrote and edited the manuscript. All authors have reviewed the manuscript and approved it for publication.

## Conflict of Interest Statement

The authors declare that the research was conducted in the absence of any commercial or financial relationships that could be construed as a potential conflict of interest.

## Footnotes

1 https://portal.gdc.cancer.gov/

2 https://david.ncifcrf.gov/home.jsp
